# Supplementary material for: Factors associated with violent offenders with mental illness in forensic psychiatric evaluations
Source: PLoS One. 2025 Dec 17;20(12):e0336918. doi: 10.1371/journal.pone.0336918 (PMC12711024; doi:10.1371/journal.pone.0336918)
Supplement: S1 File — (DOCX) [file pone.0336918.s001.docx]

**Table S1 Comparison of demographic factors between homicide offenders and theft offenders**

|  | Homicide offender (n=35) | | Theft offender  (n=198) | |  |  |
| --- | --- | --- | --- | --- | --- | --- |
|  | Mean | SD | Mean | SD | t | p value |
| Age | 39.94 | 12.65 | 39.60 | 11.63 | 0.161 | 0.873 |
|  | n | % | n | % | χ^2^ | p value |
| Male gender | 31 | 88.6 | 131 | 66.2 | 7.050 | 0.009 |
| Incomplete national education | 9 | 25.7 | 32 | 16.2 | 1.872 | 0.226 |
| Unemployed | 28 | 80.0 | 187 | 94.4 | 8.705 | 0.009 |
| Marital status |  |  |  |  |  |  |
| Single | 34 | 97.1 | 186 | 93.9 | 0.579 | 0.698 |
| Separated | 0 | 0 | 0 | 0 | - | - |
| Married | 1 | 2.9 | 12 | 6.1 | 0.579 | 0.698 |

**Table S2 Comparison of clinical factors between homicide offenders and theft offenders**

|  | Homicide offender | | Theft offender | |  |  |
| --- | --- | --- | --- | --- | --- | --- |
|  | (n=35) | | (n=198) | |  |  |
|  | n | % | n | % | χ^2^ | p value |
| Family history |  |  |  |  |  |  |
| Severe mental illness | 4 | 11.4 | 28 | 14.1 | 0.185 | 0.795 |
| Schizophrenia spectrum and other  psychotic disorders | 3 | 8.6 | 24 | 12.1 | 0.366 | 0.775 |
| Bipolar and other related disorder | 1 | 2.9 | 5 | 2.5 | 0.013 | 1.000 |
| Depressive disorder | 2 | 5.7 | 22 | 11.1 | 0.938 | 0.545 |
| Substance-related and addictive  disorders | 6 | 17.1 | 32 | 16.2 | 0.021 | 0.809 |
| Intellectual disability | 2 | 5.7 | 9 | 4.5 | 0.090 | 0.673 |
| Suicide history | 8 | 22.9 | 19 | 9.6 | 5.105 | 0.040 |
| No prior psychiatric evaluation | 8 | 22.9 | 15 | 7.6 | 7.807 | 0.011 |
| Poor treatment adherence | 35 | 100 | 186 | 93.9 | 2.236 | 0.222 |

**Table S3 Comparison of forensic psychiatric evaluation–related factors between homicide offenders and theft offenders**

|  | Homicide offender | | Theft offender | |  |  |
| --- | --- | --- | --- | --- | --- | --- |
|  | (n=35) | | (n=198) | |  |  |
|  | n | % | n | % | χ^2^ | p value |
| History of violent crimes | 10 | 28.6 | 33 | 16.7 | 2.801 | 0.102 |
| Forensic diagnosis | | |  |  |  |  |
| Severe mental illness | 10 | 28.6 | 100 | 50.5 | 5.741 | 0.018 |
| Schizophrenia spectrum and  other psychotic disorders | 9 | 25.7 | 84 | 42.4 | 3.463 | 0.091 |
| Bipolar and other related  disorder | 1 | 2.9 | 16 | 8.1 | 1.200 | 0.481 |
| Depressive disorder | 6 | 17.1 | 23 | 11.6 | 0.834 | 0.403 |
| Substance-related and addictive  disorders | 12 | 37.1 | 14 | 22.2 | 3.583 | 0.086 |
| Neurocognitive disorders | 2 | 5.7 | 16 | 8.1 | 0.234 | 1.000 |
| Intellectual disability | 5 | 14.3 | 40 | 20.2 | 0.668 | 0.493 |
| Personality disorders | 1 | 2.9 | 4 | 2.0 | 0.099 | 0.560 |

**Table S4 Association between static and dynamic crime factors and homicide and theft offenders**

|  | Homicide offender | |  |
| --- | --- | --- | --- |
|  | OR | 95% CI |  |
| Static factors |  |  |  |
| Age | 1.01 | 0.98-1.05 |  |
| Male gender | 3.44 | 1.04-11.38 |  |
| Incomplete national education | 1.89 | 0.70-5.05 |  |
| Family history of severe mental illness | 0.74 | 0.21-2.66 |  |
| No prior psychiatric evaluation | 5.75 | 1.04-31.77 |  |
| History of violent crimes | 3.19 | 1.03-9.89 |  |
| Dynamic factors | | 1.06 | 0.39-2.88 |
| Unemployed status |  |  |  |
| Single status | 0.30 | 0.09-0.99 |  |
| Severe mental illness | 1.16 | 0.12-11.67 |  |
| Substance-related and addictive disorders | 0.16 | 0.04-0.68 |  |
| Poor treatment adherence | 0.44 | 0.08-2.27 |  |
| Hosmer-Lemeshow goodness-of-fit test p value= 0.537 |  |  |  |
| OR, odds ratio; CI, confidence interval |  |  |  |

**Table S5 Moderation model analysis**

|  | Coefficient | SE | Z | p value | 95% CI |
| --- | --- | --- | --- | --- | --- |
| Gender on the association between severe mental illness and homicide crime | | | | | |
| Severe mental illness | -16.06 | 840.96 | -0.02 | 0.98 | -1664.30–1632.18 |
| Gender | 0.62 | 0.66 | 0.94 | 0.35 | -0.68–1.92 |
| Gender × Severe mental illness | 14.60 | 840.96 | 0.02 | 0.99 | -1633.64–1662.85 |
| Treatment adherence on the association between severe mental illness and homicide crime | | | | | |
| Severe mental illness | -1.14 | 1881.13 | -0.0006 | 0.9995 | -3688.09–3685.81 |
| Treatment adherence | 14.63 | 1417.72 | 0.01 | 0.9918 | -2764.05–2793.31 |
| Treatment adherence × Severe mental illness | -0.64 | 1881.13 | -0.0003 | 0.9997 | -3687.59–3686.32 |
| History of violent crime on the association between severe mental illness and homicide crime | | | | | |
| Severe mental illness | -1.95 | 0.80 | -2.43 | 0.02 | -3.53–-0.38 |
| History of violent crime | -0.13 | 0.65 | -0.20 | 0.85 | -1.40–1.15 |
| History of violent crime × Severe mental illness | 0.55 | 1.00 | 0.55 | 0.58 | -1.42–2.52 |
| Substance-related and addictive disorders on the association between severe mental illness and homicide crime | | | | | |
| Severe mental illness | -1.98 | 1.10 | -1.81 | 0.07 | -4.14–0.17 |
| Substance-related and addictive disorders | -0.90 | 0.92 | -0.97 | 0.33 | -2.71–0.91 |
| Substance-related and addictive disorders × Severe mental illness | -0.37 | 1.44 | 0.25 | 0.80 | -2.46–3.20 |

SE, standard error; CI, confidence interval
