# Supplementary material for: Factors associated with violent offenders with mental illness in forensic psychiatric evaluations
Source: PLoS One. 2025 Dec 17;20(12):e0336918. doi: 10.1371/journal.pone.0336918 (PMC12711024; doi:10.1371/journal.pone.0336918)
Supplement: Attachment — Submitted filename: Rebuttal letter 20250720.docx [file pone.0336918.s005.docx]

Dear Editors,

Thank you very much for your time and the opportunity to revise our manuscript entitled “Factors associated with violent offenders with mental illness in forensic psychiatric evaluations.” We greatly appreciate the constructive comments and suggestions provided by you and the reviewers, which have been very helpful in improving the quality of our manuscript.

We have carefully revised the manuscript in accordance with the reviewer’s comments. Below, we provide detailed responses to each point raised.

Additional Requirement:

1.Please ensure that your manuscript meets PLOS ONE's style requirements, including those for file naming. The PLOS ONE style templates can be found at

https://journals.plos.org/plosone/s/file?id=wjVg/PLOSOne_formatting_sample_main_body.pdf and https://journals.plos.org/plosone/s/file?id=ba62/PLOSOne_formatting_sample_title_authors_affiliations.pdf

Response: Thank you for the reminding. We have checked the format of out manuscript.

2. Please include a complete copy of PLOS’ questionnaire on inclusivity in global research in your revised manuscript. Our policy for research in this area aims to improve transparency in the reporting of research performed outside of researchers’ own country or community. The policy applies to researchers who have travelled to a different country to conduct research, research with Indigenous populations or their lands, and research on cultural artefacts. The questionnaire can also be requested at the journal’s discretion for any other submissions, even if these conditions are not met. Please find more information on the policy and a link to download a blank copy of the questionnaire here: https://journals.plos.org/plosone/s/best-practices-in-research-reporting. Please upload a completed version of your questionnaire as Supporting Information when you resubmit your manuscript.

Response: We have completed the questionnaire and attached the information over the end part of the manuscript.

3. Thank you for stating the following in your Competing Interests section:

“nil”

Please complete your Competing Interests on the online submission form to state any Competing Interests. If you have no competing interests, please state "The authors have declared that no competing interests exist.", as detailed online in our guide for authors at http://journals.plos.org/plosone/s/submit-now

This information should be included in your cover letter; we will change the online submission form on your behalf.

Response: Sure. Thanks.

4. We note that you have indicated that there are restrictions to data sharing for this study. PLOS only allows data to be available upon request if there are legal or ethical restrictions on sharing data publicly. For more information on unacceptable data access restrictions, please see http://journals.plos.org/plosone/s/data-availability#loc-unacceptable-data-access-restrictions.

Before we proceed with your manuscript, please address the following prompts:

a) If there are ethical or legal restrictions on sharing a de-identified data set, please explain them in detail (e.g., data contain potentially identifying or sensitive patient information, data are owned by a third-party organization, etc.) and who has imposed them (e.g., a Research Ethics Committee or Institutional Review Board, etc.). Please also provide contact information for a data access committee, ethics committee, or other institutional body to which data requests may be sent.

b) If there are no restrictions, please upload the minimal anonymized data set necessary to replicate your study findings to a stable, public repository and provide us with the relevant URLs, DOIs, or accession numbers. For a list of recommended repositories, please see

https://journals.plos.org/plosone/s/recommended-repositories. You also have the option of uploading the data as Supporting Information files, but we would recommend depositing data directly to a data repository if possible.

We will update your Data Availability statement on your behalf to reflect the information you provide.

Response: Sure. We had uploaded the de-identified data onto the site *The Qualitative Data Repository* <https://qdr.syr.edu/> The DOI is <https://doi.org/10.5064/F6JZOYI6>. Please also refer to the site <https://data.qdr.syr.edu/dataset.xhtml?persistentId=doi%3A10.5064/F6JZOYI6>

5. Please include your tables as part of your main manuscript and remove the individual files. Please note that supplementary tables (should remain/ be uploaded) as separate "supporting information" files

Response: Sure. We have included the tables into our manuscript.

6. If the reviewer comments include a recommendation to cite specific previously published works, please review and evaluate these publications to determine whether they are relevant and should be cited. There is no requirement to cite these works unless the editor has indicated otherwise.

Response: The reviewer did not mention some citations of published works.

**Reviewer #1**

1. All of the above statistical design and results appear to be appropriate, but I was confused as to why all of the description in the Methods was written in future tense instead of past tense.

Response: Thank you for the reminding. We have changed the future-tense descriptions in the method section to past-tense uses. Kindly refer to lines 220-234 on page 11.

“This study includes both continuous and categorical variables across demographic, clinical, and forensic evaluation-related factors. Continuous variables were reported as means and standard deviations, while categorical variables were expressed as frequencies and percentages. Bivariate analyses were conducted using independent sample t-tests for continuous variables and chi-square tests for categorical variables. Multiple logistic regression was performed to examine associations between static and dynamic crime factors (independent variables) and offender classification (violent vs. non-violent) as the dependent variable. Independent variables were entered into the statistical model using the Enter method. Model fit in logistic regression analysis were assessed using the Hosmer–Lemeshow goodness-of-fit test. Statistical significance was set at p < 0.05, with all tests conducted as two-tailed analyses.

To assess whether the static factors—male gender and history of violent crime—and the dynamic factors—poor treatment adherence and diagnoses from forensic psychiatric evaluation of substance-related and addictive disorders—moderate the relationship between severe mental illness and violent crime, a moderation analysis was conducted using Hayes’ PROCESS macro in SPSS [36]. Each factor was examined individually, with other factors included as covariates to control for potential confounding effects.”

2. Their conclusion is “These findings indicate that offenders referred for forensic psychiatric evaluation due to violent crime are not necessarily those with severe mental illness.” Although they have some interesting neurochemical speculation on the reason for violent crime in their sample, I did not see an adequate rebuttal to the papers in their literature that DID find an association of major mental illness with violent crime.

Response: Thank you for your kind reminder. To enhance the alignment with the reviewed literature, we have revised the relevant descriptions in the Discussion section. Kindly refer to lines 295-302 on page 17.

“In the preceding section, we reviewed large-scale database studies from Western countries [9,13], which have suggested that individuals with serious mental illness may be at higher risk of involvement in violent crime compared to the general population. However, our study did not use a general population comparison group. Instead, we focused on differentiating violent and non-violent offenders within a forensic sample. Our analysis revealed that serious mental illness was not significantly associated with violent offending in this offender population. This finding suggests that, among individuals who have already committed crimes, other risk factors beyond serious mental illness may play a more prominent role in distinguishing violent offenders from non-violent ones.”
